# The Effect of Race and Shear Stress on CRP-Induced Responses in Endothelial Cells

**DOI:** 10.1155/2021/6687250

**Published:** 2021-12-02

**Authors:** Chenyi Ling, Marc D. Cook, Heather Grimm, Maitha Aldokhayyil, Dulce Gomez, Michael Brown

**Affiliations:** ^1^Department of Kinesiology & Nutrition, University of Illinois at Chicago, Chicago, IL 60607, USA; ^2^Department of Biology & Wildlife, University of Alaska Fairbanks, Fairbanks, AK 99775, USA; ^3^Department of Kinesiology, North Carolina A&T State University, Greensboro, NC 27411, USA; ^4^Department of Exercise Science, King's College, Wilkes-Barre, PA 18711, USA; ^5^School of Kinesiology, Auburn University, Auburn, AL 36849, USA

## Abstract

**Background:**

C-reactive protein (CRP) is an independent biomarker of systemic inflammation and a predictor of future cardiovascular disease (CVD). More than just a pure bystander, CRP directly interacts with endothelial cells to decrease endothelial nitric oxide synthase (eNOS) expression and bioactivity, decrease nitric oxide (NO) production, and increase the release of vasoconstrictors and adhesion molecules. Race is significantly associated with CRP levels and CVD risks. With aerobic exercise, the vessel wall is exposed to chronic high laminar shear stress (HiLSS) that shifts the endothelium phenotype towards an anti-inflammatory, antioxidant, antiapoptotic, and antiproliferative environment. Thus, the purpose of this study was to assess the racial differences concerning the CRP-induced effects in endothelial cells and the potential role of HiLSS in mitigating these differences.

**Methods:**

Human umbilical vein endothelial cells (HUVECs) from four African American (AA) and four Caucasian (CA) donors were cultured and incubated under the following conditions: (1) static control, (2) CRP (10 *μ*g/mL, 24 hours), (3) CRP receptor (Fc*γ*RIIB) inhibitor followed by CRP stimulation, (4) HiLSS (20 dyne/cm^2^, 24 hours), and (5) HiLSS followed by CRP stimulation.

**Results:**

AA HUVECs had significantly higher Fc*γ*RIIB receptor expression under both basal and CRP incubation conditions. Blocking Fc*γ*RIIB receptor significantly attenuated the CRP-induced decrements in eNOS expression only in AA HUVECs. Finally, HiLSS significantly counteracted CRP-induced effects.

**Conclusion:**

Understanding potential racial differences in endothelial function is important to improve CVD prevention. Our results shed light on Fc*γ*RIIB receptor as a potential contributor to racial differences in endothelial function in AA.

## 1. Introduction

Endothelial dysfunction plays an integral role in the progression of cardiovascular disease (CVD) and is characterized by a reduction in nitric oxide (NO) that promotes a proinflammatory and proatherogenic environment [1–4]. It is well documented that African Americans (AA), regardless of admixture, exhibit a higher prevalence, an earlier onset, and greater severity of CVD [5], with approximately twice the rate in essential hypertension (HT) compared to Caucasians (CA) [6]. Furthermore, the elevated levels of systemic inflammation and endothelial dysfunction observed in AAs compared to CAs may contribute to the higher prevalence of cardiovascular risk factors and the pathogenesis of HT in this population.

Several large-scale studies, including the Dallas Heart Study, have reported racial differences in plasma C-reactive protein (CRP) levels, with AA exhibiting significantly higher median CRP levels compared to CA [7]. Current evidence has demonstrated that plasma CRP level is not only a prognostic biomarker of CVD but also has a direct effect on endothelial cells (ECs) by reducing endothelial NO synthase (eNOS) mRNA and protein expression necessary for NO production [4, 8–10]. CRP has also been associated with endothelial dysfunction, exhibited as diminished large artery compliance in AA but not in CA [11].

Furthermore, gene knock-out and pharmacological inhibition experiments have provided evidence that CRP effects are mediated by Fc*γ*RIIB (CD32) and Fc*γ*RI (CD64) receptors [12–14]. Tanigaki et al. showed that CRP leads to the phosphorylation of Fc*γ*RIIB's cytoplasmic ITIM that in turn results in the recruitment and activation of SHIP1 in endothelium to attenuate the downstream PI3K signaling and inactivation of eNOS [15, 16]. The Fc*γ*RIIB receptor is necessary for the CRP antagonism of eNOS, exhibited in blocking IgG antibodies preventing the CRP-induced effects.

Laminar shear stress (LSS), a frictional force exerted by the blood on the endothelium, is a major determinant of vessel diameter [17, 18] and vascular remodeling [19, 20]. Aerobic exercise elevates LSS, compared to resting physiological levels [21, 22]. In addition, chronic exposure to high LSS (HiLSS; > 10 dyne/cm^2^) shifts EC phenotype towards an anti-inflammatory, antioxidant, antiapoptotic, and antiproliferative environment [23, 24]. Other benefits of HiLSS include a robust increase in eNOS protein expression and NO production [25–27] unlike static conditions and resting physiological levels of shear stress (0-4 dyne/cm^2^), which have the opposite effects [28].

Racial differences in endothelial function are well established. Yet, research in this area has largely been conducted at the physiologic level. Mechanistic research on the effect of CRP on the endothelium is still lacking. Therefore, the purpose of this study was to assess CRP as a potential mediator of racial differences in endothelial function. Additionally, examine the effects of HiLSS using primary ECs from AA and CA donors.

## 2. Materials and Methods

### 2.1. Cell Culture

Primary human umbilical vein ECs (HUVECs) were purchased from Lonza Walkersville Inc. (Walkersville, MD) and chosen for this study to obtain normal, naive ECs and minimize the possibility of preexistent factors influencing endothelial function (age, CVD risk factor exposure, etc.) [29]. Cells from four AA and four CA donors were cultured and treated under identical conditions, and experiments were performed at 80-90% confluency in duplicates.

Ethical concerns pertaining to human cell culture work are important and not overlooked by our laboratory. The University of Illinois at Chicago Office for the Protection of Research Subjects determined that this work does not meet the definition of human subject research as defined by 45 CFR 46.102(f). Further, Lonza Walkersville, Inc. accepts tissue only if consent for research has been obtained. Audits are frequently conducted to ensure appropriate operational procedures, and compliance with the Protection of Human Subjects regulations, of consent processes and receipt of any de-identified demographic or medical history information from donors.

### 2.2. Laminar Shear Stress Protocol

Confluent monolayers were exposed to an arterial level of unidirectional HiLSS (20 dyne/cm^2^) for 24 hours with a rotating cone-in-plate instrument at a 0.5° angle, designed for 100 mm tissue culture dish [26, 30]. Using a cone-and-plate device provides a unidirectional laminar flow with a moving upper conical boundary and thus does not generate any pressure gradients that could alter cell function [31, 32].

### 2.3. Materials

Recombinant human CRP was purchased from EDM Millipore. The known effects of CRP on endothelial cells have been ascribed to endotoxins and Azide. All CRP preparations were purified under sterile conditions using endotoxin-removal columns (Pierce Biochemicals), and CRP was used only if the concentration of endotoxin was ≤0.125 EU/mL. All cell culture media was endotoxin-free. Dialysis was used for Azide removal using 1 liter of PBS buffer with the buffer being changed three times. In 4 major cohort studies performed in the United States, the quintile distributions of CRP for men and for women not taking hormone replacement were < 0.5, 0.5 to 1.0, 1.0 to 2.0, 2.0 to 4.0, and > 4.0 mg/L [33], with a 10 *μ*g/mL (10 mg/L) level indicative of an acute infection or trauma [33]. Therefore, a 10 *μ*g/mL dose of CRP was used for 24 hours to examine the CRP-induced effects in these experiments. For inhibitory experiments, HUVECs were pretreated with a concentration of 100 *μ*g/mL of Fc*γ*RIIB antibody (AB; IgG) from R&D Systems (Minneapolis, MN) for 1 hr. This dose was chosen based on preliminary serial concentration experiments.

### 2.4. Western Blot

Immediately following treatment, both the static and HiLSS culture dishes were harvested for protein analysis as previously described [34]. Aliquots of cell lysate were separated by NuPAGE (Bis-Tris) gels and transferred to polyvinylidene fluoride (PVDF) membranes, which were blocked with 5% nonfat dry milk dissolved in Tris-Buffered Saline and then incubated overnight with primary antibodies at 4°C. Immunoreactive proteins were detected by chemiluminescence with Thermo Scientific SuperSignal (Pierce Biotechnology, IL). Primary antibodies included anti-eNOS, Fc*γ*RIIB, and anti-*β*-actin (Santa Cruz Biotechnology, CA). Western blot densitometry analyses were completed using the ImageJ software to quantify protein expression levels. Western blot data are represented as bar graphs to reflect the relative expression normalized to *β*-actin protein expression, which was used as an internal control.

### 2.5. Nitrate/Nitrite (NOx) Measurements

Following treatment, cell supernatant was collected and stored immediately at –80°C. NOx assay kit (abcam, Cambridge, MA) was used to measure NOx levels. The NOx colorimetric kit utilizes total nitrate/nitrite (NO end-products) quantification via Griess reagent. This method has been validated to reflect NOx in biological samples with the intra/interassay CV < 5% for NOx in the observed ranges [35].

### 2.6. Statistical Analysis

All variables were checked for normal distribution with the Shapiro-Wilk test, and descriptive statistics were performed. A one-way (within race/ethnicity and within experimental conditions) and two-way ANOVA was used to examine any race by shear stress interaction effects. Post hoc adjustments for multiple comparisons were done using the Bonferroni's test. Analysis was performed using SPSS version 21.0 (SPSS Inc., Chicago, IL). Data are expressed as mean ± SE and the level of significance set at *p* ≤ 0.05.

## 3. Results

**Figure 1:**
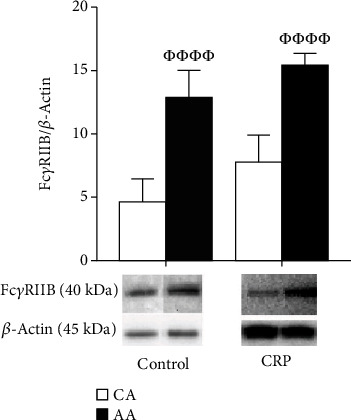
Racial difference in Fc*γ*RIIB receptor protein expression. HUVECs from CA and AA were incubated with CRP (10 *μ*g/mL) for 24 hr (mean ± SE). *ΦΦΦΦp* < 0.001, compared to CA.

**Figure 2:**
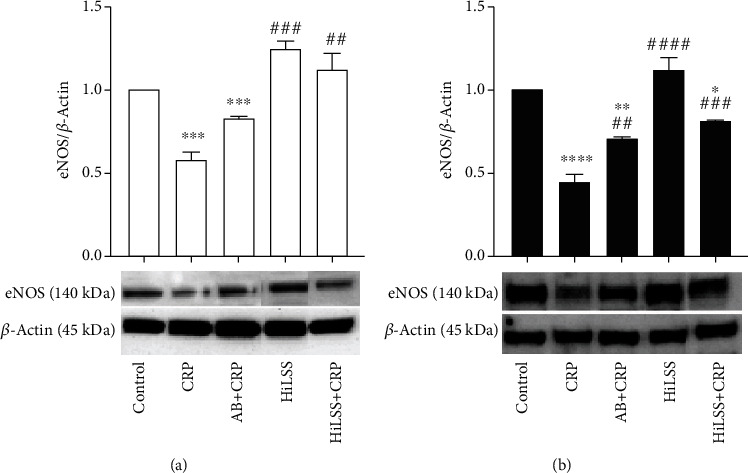
eNOS protein expression under different conditions. Effects of HiLSS application and CRP incubation were evaluated in (a) CA HUVECs and (b) AA HUVECs (mean ± SE). ^∗^*p* < 0.05, ^∗∗^*p* < 0.01, ^∗∗∗^*p* < 0.005, ^∗∗∗∗^*p* < 0.001, compared to control. ## *p* < 0.01, ### *p* < 0.005, #### *p* < 0.001, compared to CRP.

**Figure 3:**
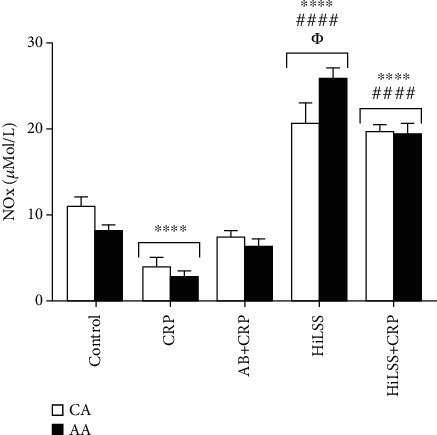
Nitrate/nitrite (NOx) levels under different conditions in CA and AA HUVECs (mean ± SE). ^∗∗∗∗^*p* < 0.001, compared to control. #### *p* < 0.001, compared to CRP. *Φp* < 0.05, between racial groups.

Under basal and CRP conditions, AA HUVECs expressed significantly higher levels of Fc*γ*RIIB receptors than CA (Figure 1, *p* < 0.001). Blocking Fc*γ*RIIB receptor attenuated CRP-induced racial difference in eNOS protein expression (Figure 2, *p* < 0.01). CRP significantly reduced NOx production in AA and CA HUVECs (Figure 3, *p* < 0.001). Blocking the Fc*γ*RIIB receptor reduced the CRP-induced blunting of NOx production, but the change was not statistically significant.

**Figure 4:**
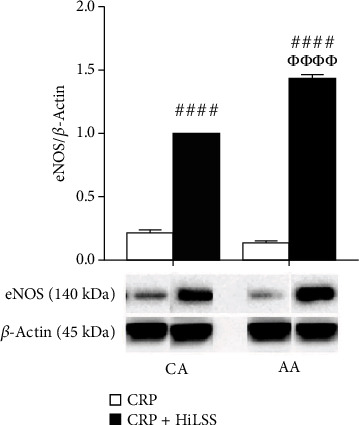
The effect of HiLSS on eNOS protein expression after CRP preincubation. HUVECs from CA and AA were preincubated with CRP (10 *μ*g/mL) for 24 hr followed by static control or HiLSS (20 dyne/cm^2^) for another 24 hr (mean ± SE). #### *p* < 0.001, compared to CRP. *ΦΦΦΦp* < 0.001, compared to CA.

**Figure 5:**
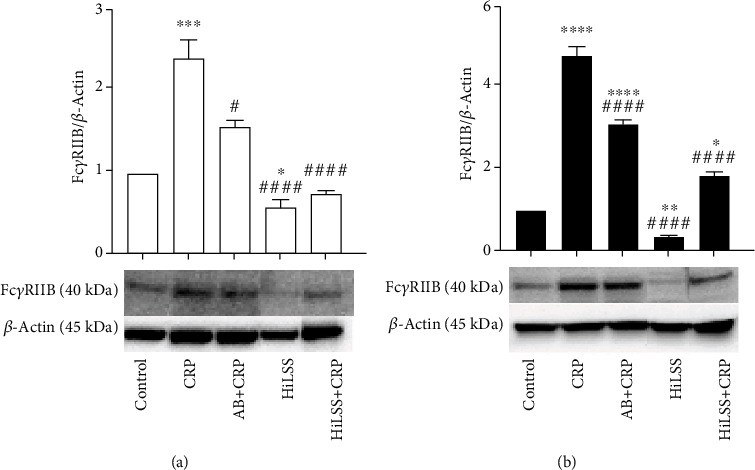
Fc*γ*RIIB receptor protein expression under different conditions. Effects of HiLSS application and CRP incubation were evaluated in (a) CA HUVECs and (b) AA HUVECs (mean ± SE). ^∗^*p* < 0.05, ^∗∗^*p* < 0.01, ^∗∗∗^*p* < 0.005, ^∗∗∗∗^*p* < 0.001, compared to control. # *p* < 0.05, ## *p* < 0.01, #### *p* < 0.001, compared to CRP.

HiLSS significantly mitigated CRP-induced attenuation of eNOS expression in both racial groups (Figure 4, *p* < 0.001). Within each racial group, applying HiLSS prior to CRP incubation resulted in the greatest attenuation of CRP induced effects on eNOS (Figure 2, CA: *p* < 0.01, AA: *p* < 0.005). Moreover, a robust downregulation in the expression of Fc*γ*RIIB receptor was observed following HiLSS (Figure 5, *p* < 0.001). Exposing HUVECs to HiLSS prior to CRP incubation attenuated CRP-induced upregulation of Fc*γ*RIIB receptor (Figure 5, *p* < 0.001). Furthermore, only HiLSS condition resulted in higher production in NOx in the culture media in AA compared to CA (Figure 3, *p* < 0.05).

## 4. Discussion

The key findings of this study suggest that HUVECs obtained from AA and CA donors respond differently to CRP, with AA HUVECs exhibiting higher Fc*γ*RIIB receptor expression under basal and CRP conditions, and being more responsive to HiLSS than CA. HiLSS reversed the detrimental effects of CRP on eNOS in ECs. Furthermore, Fc*γ*RIIB receptor mediates the CRP-induced detrimental effects on endothelial function. To our knowledge, this is the first study to evaluate racial differences in CRP-induced effects in primary ECs and the effect of HiLSS.

Numerous epidemiological studies provide solid evidence that serum CRP levels are higher in AA compared to CA with similar CVD risks [7, 36, 37]. The inverse association of CRP and endothelial function was first shown when acetylcholine-induced brachial artery dilation was reduced in patients with elevated levels of serum CRP [38, 39] suggesting that CRP might affect vasodilatory capacity, potentially by affecting NO bioavailability. NO is one of the most important vasodilators released by the endothelium, and reduced bioavailability is a hallmark of endothelial dysfunction in AA *in vitro* [40]. The importance of NO is not limited to vasodilation properties as it inhibits inflammation, platelet aggregation, and smooth muscle cell proliferation [41–43].

Inflammation largely contributes to endothelial dysfunction and reduces NO bioavailability in healthy people [44]. *In vivo*, AA have higher levels of inflammation, specifically CRP, than their CA peers in healthy [11] and diseased cohorts. AA diabetic patients with microalbuminuria, a population associated with higher levels of CRP, showed a blunted response to vasodilators due to decreased levels of NO production [38]. *In vitro* studies have shown that HUVECs obtained from AA exhibit higher oxidative stress levels, higher inflammation and adhesion molecule expression, and a greater degree of fibrinolytic potential compared to CA [40, 45, 46]. There is a reduction in NO bioavailability accompanied by an increased production of superoxide (O_2_^−^) and peroxynitrite (ONOO^−^) in AA HUVECs. If these results translate to humans, they may contribute to the higher prevalence and severity of endothelial dysfunction and hypertension in AA [40]. In addition, our group has shown that AA HUVECs produce more endothelial microparticles (an emerging biomarker of endothelial dysfunction) compared to CA HUVECs which correlates with inflammation, *in vivo* and *in vitro* [47]. In our study, AA HUVECs exhibited significantly greater expression of Fc*γ*RIIB receptor. Inferring this to humans, CRP-induced effect may converge with other mechanisms, such as oxidative stress, to promote endothelial dysfunction and vascular diseases in AA.

It has been reported that CRP binding to Fc*γ* receptors, Fc*γ*RI, and Fc*γ*RIIB on ECs decreases eNOS and prostacyclin and increases IL-8 and adhesion molecules [48]. Blocking Fc*γ*RI and Fc*γ*RIIB by antibodies or transfection with small interference RNA effectively attenuated NF-*κ*B activity and inhibited VCAM-1 and ICAM-1 upregulation [49]. Our investigation shows that blocking Fc*γ*RIIB inhibited the CRP-induced suppression of eNOS protein expression in AA HUVECs. Similar effects were found in other studies by attenuating the decrease in eNOS expression and the increase in IL-8 after CRP incubation [14, 15]. Thus, Fc*γ* receptors serve as a mediators of CRP-induced biological effects in HUVECs.

It is undeniable that social determinants contribute to racial disparities in HT and CVD. However, it is imperative to determine biological mediators of racial disparity in CVD. The current study provides preliminary evidence that higher levels of Fc*γ*RIIB receptor expression contributes to, at least partially, the greater CRP-induced biological effects in AA HUVECs. The novelty of our study is that the synergistic effects of increased CRP receptors and greater response to CRP may explain the racial differences in endothelial function. This may be particularly relevant for clinical studies involving chronic inflammation or hypertension in AA cohorts. In addition, the pre-IgG incubation eliminated, to an extent, the CRP-induced effects. Despite the functional similarities between CRP and IgG, our data suggest that the CRP detrimental effect on eNOS is only partially mediated by the Fc*γ* receptor pathways.

An important role of the endothelium in the vasculature is sensing blood flow to regulate vascular function. HiLSS induces an atheroprotective phenotype by altering EC gene expression profile and initiating endothelial quiescence [50]. DNA microarray data have shown that HiLSS (12 dyne/cm^2^, 24 hours) downregulates genes related to inflammation and proliferation in ECs [50]. On the other hand, low LSS (< 5 dyne/cm^2^) displays an atherogenic phenotype [28]. Recently, it has been shown that AA HUVECs respond differently to HiLSS, evidenced by a significantly larger reduction in NADPH oxidase subunit expression and a greater production of antioxidants [25].

Our results support the hypothesis that HiLSS can attenuate the CRP-induced proatherogenic effects and eliminate the racial differences in suppressed eNOS expression in HUVECs response to CRP. AA ECs may be more responsive to HiLSS, as evidenced by higher NOx production. In addition, the application of HiLSS prior to CRP incubation alleviated eNOS expression for both racial groups. However, compared to CA, AA HUVECs exhibited a greater recovery of eNOS. Based on our findings and others, translating this to human, it appears that the endothelium, in terms of endothelial dysfunction, AA may benefit more from high levels of blood flow generated by aerobic exercise compared to CA. Potential mechanisms remain to be investigated.

Our data also suggests that HiLSS maintains Fc*γ*RIIB receptor expression in HUVECs at lower levels compared to the static condition. The inhibitory effect of HiLSS on Fc*γ*RIIB receptor expression and function may contribute to the antiatherogenic effect of laminar flow. This may help regulate endothelial function, cell survival, and integrity under inflammatory conditions to protect the endothelium from moving toward a proatherogenic status.

### 4.1. Limitations

Although our observations are novel, the results and conclusion drawn from our study should be interpreted with caution for several reasons. Long-term influences of risk factors, social determinants of CVD, and other physiological changes cannot be applied in this cell model. This highly controlled *in vitro* model does not prove a causal relationship between higher expression of CRP receptors and CVD prevalence in AA. However, this highly controlled *in vitro* model does eliminate the uncontrolled physiological confounding variables found in human subjects. Based on the outcomes of this study, it is reasonable to suggest that greater CRP receptor expression is a potential mechanism that may explain racial differences. When compared to human studies, the sample size of our experiments is smaller, but cell culture studies inherently have less random error because it eliminates complex physiology and other confounding variables that allow for the smaller sample size to be analyzed. Previously in our lab, a sample size that has produced positive results entailed an *N* = 4 donors for each group and thus deemed appropriate to follow suit for this study [25, 47, 51, 52]. Future studies should examine Fc*γ* receptor expression in ECs isolated from human vessels where CVD is evident. Second, although we used HUVECs in our study, there are different EC types throughout the human body where Fc*γ* receptor expression and activities might differ between racial groups. Lastly, while the majority of CRP is produced systemically by the liver, there is data showing that human aortic ECs (HAECs) synthesize and secrete CRP locally [53]. However, the amount of CRP produced by ECs is relatively much less than that from the liver. We are unaware of any reports of this in HUVECs, nor did we measure CRP mRNA/protein in our experiments.

## 5. Conclusion

A better understanding of the racial differences in endothelial function will help us improve hypertension prevention and treatment strategies. Our results highlight Fc*γ*RIIB receptor as a novel therapeutic target, particularly in AA. Future studies are needed to investigate the variance of the Fc*γ*RIIB expression in other types of ECs. Additionally, there is a need for translational research to elucidate the role of different Fc*γ* receptors in AA and CA with elevated CRP levels. Uncovering the clinical consequences of Fc*γ* receptors on the racial differences in endothelial function could result in a more individualized prevention/treatment approach.

## Data Availability

The data used to support the findings of this study are available from the corresponding author upon request, after publication.
